# Activation of ATP-ubiquitin-dependent proteolysis in skeletal muscle in vivo and murine myoblasts in vitro by a proteolysis-inducing factor (PIF)

**DOI:** 10.1054/bjoc.2001.1879

**Published:** 2001-07

**Authors:** M J Lorite, H J Smith, J A Arnold, A Morris, M G Thompson, M J Tisdale

**Affiliations:** Pharmaceutical Sciences Research Institute, Aston University, Birmingham, B4 7ET, UK; Rowett Research Institute, Greenburn Road, Bucksburn, Aberdeen, AB21 9SB, UK

**Keywords:** cachexia, muscle proteolysis, proteolysis- inducing factor (PIF), ubiquitin-proteasome proteolysis

## Abstract

Loss of skeletal muscle is a major factor in the poor survival of patients with cancer cachexia. This study examines the mechanism of catabolism of skeletal muscle by a tumour product, proteolysis-inducing factor (PIF). Intravenous administration of PIF to normal mice produced a rapid decrease in body weight (1.55 ± 0.12 g in 24 h) that was accompanied by increased mRNA levels for ubiquitin, the Mr 14 000 ubiquitin carrier-protein, E2, and the C9 proteasome subunit in gastrocnemius muscle. There was also increased protein levels of the 20S proteasome core and 19S regulatory subunit, detectable by immunoblotting, suggesting activation of the ATP-ubiquitin-dependent proteolytic pathway. An increased protein catabolism was also seen in C_2_C_12_ myoblasts within 24 h of PIF addition with a bell-shaped dose–response curve and a maximal effect at 2–4 nM. The enhanced protein degradation was attenuated by anti-PIF antibody and by the proteasome inhibitors MG115 and lactacystin. Glycerol gradient analysis of proteasomes from PIF-treated cells showed an elevation in chymotrypsin-like activity, while Western analysis showed a dose-related increase in expression of MSSI, an ATPase that is a regulatory subunit of the proteasome, with a dose–response curve similar to that for protein degradation. These results confirm that PIF acts directly to stimulate the proteasome pathway in muscle cells and may play a pivotal role in protein catabolism in cancer cachexia. © 2001 Cancer Research Campaign http://www.bjcancer.com


British Journal of Cancer (2001) **85**(2), 297–302
© 2001 Cancer Research Campaign
doi: 10.1054/ bjoc.2001.1879, available online at http://www.idealibrary.com on IDEAL®

http://www.bjcancer.com

### Activation of ATP-ubiquitin-dependent proteolysis in skeletal muscle in vivo and murine myoblasts in vitro by a proteolysis-inducing factor (PIF)

**MJ Lorite[1], HJ Smith[1], JA Arnold[1], A Morris[2], MG Thompson[2] and MJ Tisdale[1]**

[1]Pharmaceutical Sciences Research Institute, Aston University, Birmingham B4 7ET, UK; [2]Rowett Research Institute, Greenburn Road, Bucksburn, Aberdeen AB21 9SB, UK

**Summary** Loss of skeletal muscle is a major factor in the poor survival of patients with cancer cachexia. This study examines the mechanism of catabolism of skeletal muscle by a tumour product, proteolysis-inducing factor (PIF). Intravenous administration of PIF to normal mice produced a rapid decrease in body weight (1.55 ± 0.12 g in 24 h) that was accompanied by increased mRNA levels for ubiquitin, the Mr 14 000 ubiquitin carrier-protein, E2, and the C9 proteasome subunit in gastrocnemius muscle. There was also increased protein levels of the 20S proteasome core and 19S regulatory subunit, detectable by immunoblotting, suggesting activation of the ATP-ubiquitin-dependent proteolytic pathway. An increased protein catabolism was also seen in $C_2C_{12}$ myoblasts within 24 h of PIF addition with a bell-shaped dose–response curve and a maximal effect at 2–4 nM. The enhanced protein degradation was attenuated by anti-PIF antibody and by the proteasome inhibitors MG115 and lactacystin. Glycerol gradient analysis of proteasomes from PIF-treated cells showed an elevation in chymotrypsin-like activity, while Western analysis showed a dose-related increase in expression of MSSI, an ATPase that is a regulatory subunit of the proteasome, with a dose–response curve similar to that for protein degradation. These results confirm that PIF acts directly to stimulate the proteasome pathway in muscle cells and may play a pivotal role in protein catabolism in cancer cachexia. © 2001 Cancer Research Campaign http://www.bjcancer.com

**Keywords**: cachexia; muscle proteolysis; proteolysis- inducing factor (PIF); ubiquitin-proteasome proteolysis

Muscle wasting is an important component of the process of cachexia. In a study of lung cancer patients who had lost 30% of their pre-illness stable weight, there was a 13% decrease in lean body mass, when compared to a group of controls matched for age, sex, height and pre-illness stable weight (Preston et al, 1987). This overall change reflected a 75% decrease in skeletal muscle protein, but with no change in visceral protein mass. This depletion of skeletal muscle mass would lead to significant impairment of respiratory function (Windsor and Hill, 1988), followed by death of the patient from hypostatic pneumonia. Thus in order to devise effective therapy for this condition it is necessary to understand the mechanism by which selective depletion of skeletal muscle occurs.

A number of studies have provided evidence for an increased whole-body protein turnover in patients with lung (Heber et al, 1982), colon (Fearon et al, 1988) and hepatocellular carcinoma (O'Keefe et al, 1990). This may be due to a depression in protein synthesis (Eden et al, 1984), an increase in protein degradation (Lundholm et al, 1976) or a combination of the 2. A depression in muscle protein synthesis was observed in weight-losing cancer patients without a change in total body protein synthesis or degradation (Eden et al, 1984). In these patients muscle protein synthesis only accounted for 8% of total body protein synthesis compared with 53% for healthy controls. Thus the maintenance of the total protein synthetic rate in these patients appears to be due to a 2-fold increase in non-skeletal muscle protein synthesis, most likely arising from an increased hepatic production of acute phase proteins (Fearon et al, 1991).

We have recently isolated a proteolysis-inducing factor (PIF), both from a cachexia-inducing murine tumour (Todorov et al, 1996a), and from the urine and tumours of patients with cancer cachexia and a range of tumour types (Cariuk et al, 1997). Administration of PIF to normal mice produced a 10% loss of overall body weight in 24 h, with selective depletion of lean body mass (Todorov et al, 1996a; Cariuk et al, 1997). Soleus and gastrocnemius muscle weights decreased, while kidney and heart remained unchanged (Lorite et al, 1998). The mechanism by which PIF induces protein degradation in skeletal muscle is not known, but previous studies have shown this to be an energy-dependent process, with no contribution from either the lysosomal or calcium dependent proteolytic pathways (Lorite et al, 1998). In cachectic mice bearing the MAC16 tumour, there is an enhanced expression of the components of the ATP-ubiquitin-dependent proteolytic pathway (Lorite et al, 1998). Since PIF has been shown to be responsible for the cachexia in this model, this study reports changes in the expression of key components of this pathway in mice administered PIF. To confirm that PIF exerts a direct effect on the pathway parallel studies have been carried out in $C_2C_{12}$ murine myoblasts in vitro. This surrogate model system was

*Received 23 January 2001*
*Revised 30 March 2001*
*Accepted 2 April 2001*

*Correspondence to:* M Tisdale chosen because we have previously shown (Smith et al, 1999) myoblasts to undergo an increased protein degradation in the presence of PIF.

### MATERIALS AND METHODS

### Animals

Pure strain female NMRI mice (18–20 g) were obtained from our own breeding colony and were fed a rat and mouse diet (Special Diet Services, Witham, Essex, United Kingdom) and water ad libitum. Fragments of the MAC16 tumour, excised from donor animals with established weight loss, were implanted into the flanks of NMRI mice (20–25 g) by means of a trocar as described (Beck and Tisdale, 1987). Tumours were excised from mice with weight loss between 20 and 25% and used to purify PIF, as agreed by the Coordinating Committee on Cancer Research of the United Kingdom for the welfare of animals with neoplasms.

### Purification of PIF

PIF was purified from solid MAC16 tumours by homogenization and precipitation of the protein with ammonium sulfate (40% w/v). The supernatant formed by centrifugation at 4000 ***g*** was subjected to affinity chromatography using a monoclonal antibody immobilized to a protein A matrix as described (Todorov et al, 1996b). The immunogenic fractions were concentrated and used without further purification, since the major contaminant was PIF bound to albumin.

### Isolation of RNA and Northern blot analysis

Solid MAC16 tumours were excised from donor NMRI mice with weight loss between 20 and 25% and were used to purify PIF using affinity chromatography as described (Todorov et al, 1996b). Female NMRI mice (20 g) were injected into the tail vein with either PIF (7–10 µg in 100 µl PBS) or PBS as a control, 4 times at 2.5 h intervals starting at 10.00 h (Todorov et al, 1996a; Cariuk et al, 1997). After 24 h from the first injection the animals were terminated and total RNA was extracted from gastrocnemius muscle and heart using the acid guanidinium isothiocyanate/phenol/chloroform-isoamylalcohol method (Chomczynski and Sacchi, 1987) and quantitated by absorbance at 260/280 nm. Total RNA (10 µg) was heat-denatured at 65°C for 10 min and electrophoresed in 1.2% (w/v) agarose gels containing 0.67% formaldehyde. The RNA was electrophoretically transferred onto a nylon membrane (Gene Screen, NEN Research Products, MA, USA) and covalently linked to the membrane using a Spectrolinker XL-1000 UV cross-linker (120 000 mJ $cm^{-2}$). Membranes were prehybridized at 42°C in formamide (50%), dextran sulfate (10%), NaCl (1 M), bovine serum albumin (0.2%), polyvinylpyrolidone (0.2%), Ficol (0.2%), sodium pyrophosphate (0.1%), sodium dodecyl sulfate (1%), Tris.HCl (0.05 M, pH 7.5) and denatured salmon sperm DNA (100 µg $ml^{-1}$). The cDNA probes used were for ubiquitin (Wing and Goldberg, 1993), purchased from the American Type Culture Collection, the 14 K E2 ubiquitin carrier protein (Wing and Banville, 1994), a 0.6-kb KspI/KpnI fragment of the coding region, kindly provided by Dr Simon Wing (McGill University, Montreal, Canada), rat proteasome subunit 9 (Kumatori et al, 1990) kindly donated by Dr Walter Spevak (Boehringer Ingelheim, Vienna, Austria) and glyceraldehyde-3-phosphate dehydrogenase (GAPDH) as internal standard. The cDNA probes were labelled to high specific activity (~$10^9$ cpm µg $DNA^{-1}$) using the Amersham Megaprime DNA labelling system (Amersham, Bucks, United Kingdom). Hybridization was performed at 42°C, overnight, after which the membranes were washed twice with 30 mM trisodium citrate and 300 mM sodium chloride (2 × SSC buffer), followed by incubation twice for 1 h with SSC-1% SDS, with a final wash with 0.1 × SSC for 5 min at room temperature. Quantitation was either by instantimager recording (Packard, Berks, UK) or by densitometric scanning of autoradiographs. Relative amounts of RNA loaded in each lane were visualized by staining with ethidium bromide.

### Measurement of total protein breakdown

$C_2C_{12}$ myoblasts were seeded at 4 × $10^4$ cells per well in 2 ml Dubecco's modified Eagles medium in 6-well multidishes. After 24 h cells were labelled with L-[2,6-$^3$H] phenylalanine (10 µl per ml of medium of a stock solution containing 75 µmoles L-phenylalanine and 50 µCi L-[2,6-$^3$H] phenylalanine per ml) for 24 h. After labelling the cells were washed in PBS, pre-incubated in fresh DMEM for 2 h without phenol red (3 ml) in the presence of antibody (10 µg $ml^{-1}$) or proteasome inhibitors prior to the addition of PIF and phenylalanine (2 mM). The amount of radioactivity released into the medium during a 24 h period was measured as described (Smith et al, 1999).

### Measurement of proteasome activity

The activity of 26S proteasomes was measured according to the method of Orino et al (1991), with some modifications. $C_2C_{12}$ myoblasts were washed twice in ice-cold PBS and sonicated in 20 mM Tris. HCl, pH 7.5, 2 mM ATP, 5 mM $MgCl_2$ and 1 mM dithiothreitol by 3 pulses of 15 s with a 10s interval. The sonicate was then centrifuged for 10 min at 15 000 rpm at 4°C and the resulting supernatant used for assay. About 1 ml of the cell lysate (about 5 mg protein) was loaded onto 14 ml of a linear gradient of 10–40% (v/v) glycerol in sonicating buffer. The gradient was centrifuged at 24 000 rpm for 22 h at 4°C and 0.5 ml fractions were collected for the determination of chymotrypsin-like-activity of the proteasome. Activity was determined using the fluorogenic substrate succinyl-LLVY-MCA (0.1 mM) in 100 mM Tris. HCl, pH 8.0 and 10 µl of each fraction. The reaction was terminated by the addition of 80 mM sodium acetate and the fluorescence was measured with an excitation of 360 nm and an emission of 460 nm. The protein concentration of the sample was determined using the Bradford assay (Sigma Chemical Co, Poole, Dorset, UK). This method was also used for gastrocnemius and heart muscle extracts.

### Western blot analysis

Samples (5 µg protein) were resolved on 10% sodium dodecylsulfate, polyacrylamide gels and transferred to 0.45 µm nitrocellulose membranes (Hybond A, Amersham, UK), which had been blocked with 5% Marvel in Tris buffered saline (TBS) at 4°C overnight. The primary antibodies used were rabbit polyclonal antisera to the 20S proteasome core (Affiniti Research Products, Exeter, UK) at a dilution of 1:1000 and mouse anti-human MSSI (equivalent to the S7 regulatory subunit of the 26S proteasome (Dubiel et al, 1993) at a 1:1000 dilution. The secondary antibodies were peroxidase-conjugated, either goat anti-rabbit, (Sigma Chemical Co, Dorset,

 © 2001 Cancer Research Campaign

UK) or sheep anti-mouse (Amersham, UK) and were used at a 1:2000 dilution. Incubation was carried out for 2 h at room temperature and development was by enhanced chemiluminescence (ECL) (Amersham, UK).

### Statistical analysis

Differences between single groups were analysed by Student's *t*-test, while multiple group comparisons were made by ANOVA followed by Tuckey's test.

### RESULTS

Intravenous administration of PIF to female NMRI mice produced a progressive decrease in body weight as previously reported (Todorov et al, 1996a, 1996b; Cariuk et al, 1997). In the experiment depicted in Figures 1 and 2 the animals had lost 1.55 ± 0.12 g (8.2% of starting weight $P < 0.001$ from control) within 24 h of the first injection of PIF. This weight loss represented loss of lean body mass with a 17% decrease in wet weight of soleus muscles and a 16% decrease in protein content. Previous studies have shown a significant elevation of the high molecular mass conjugates of ubiquitin in the soluble gastrocnemius muscle proteins after treatment with PIF (Lorite et al, 1998). The expression of both transcripts of ubiquitin (+ 70% for the 1.2 kb mRNA species and + 64% for the 2.4 kb species $P < 0.05$ from control) were also increased in gastrocnemius muscle 24 h after administration of PIF (Figure 1A). This suggests that the ATP-ubiquitin-dependent proteolytic pathway was activated by PIF. However, since ubiquitin has roles other than in proteolysis (St John et al, 1986) the mRNA for other components of the pathway was investigated. The RNA blots were therefore probed with the cDNA of the Mr 14 000 E2, one of the few E2 species functioning in E3-dependent ubiquitin-conjugate formation (Wing and Banville, 1994). 2 mRNA transcripts of 1.2 and 1.8 kb were detectable with this probe arising from different sites of polyadenylation (Figure 1B). There was an increase in the mRNA levels for both transcripts in the gastrocnemius muscle of mice administered PIF, compared with control animals, with a more pronounced increase in the 1.2 kb transcript (+ 83% $P < 0.05$ from control) than the 1.8 kb transcript (+31%). This effect was specific to skeletal muscle, since heart from PIF-treated animals showed only a marginal increase in the 1.2 kb E2 transcript, and no change in the 1.8 kb transcript compared with control animals (Figure 1C). Hybridization of the RNA blots with cDNA encoding the C9 subunit of the 20S proteasome, the proteolytic core of the 26S proteasome, which degrades ubiquitin conjugates, showed almost a doubling of the 1.3 kb transcript in PIF-treated mice compared with PBS-treated controls (from 1931 ± 813 to 3866 ± 721 arbitary units in PIF-treated mice). Control experiments showed that the level of mRNA for GAPDH, a housekeeping gene unrelated to protein breakdown, and RNA loading was similar in both groups of mice, as evidenced by ethidium bromide staining of gels.

These coordinate changes in gene expression suggest that enhanced ATP-ubiquitin-dependent proteolysis was responsible for muscle protein catabolism induced by PIF. To determine whether PIF also produced increased intracellular protein levels of the proteasome complex cellular extracts from gastrocnemius muscle were Western blotted using a rabbit polyclonal antibody to the 20S proteasome core. 2 major bands were observed with molecular masses between 25 and 30 000 Daltons corresponding with subunits of the proteasomal catalytic complex (Figure 2A). When compared with control animals densitometric analysis showed a 7-fold increase in both the lower molecular weight subunit ($P <$ 0.005) and the higher molecular weight subumit ($P < 0.02$) in PIF-treated animals. Western analysis of gastrocnemius muscle of PIF-treated mice using anti-MSSI showed a 2-fold ($P < 0.01$) increase in the expression of a protein of Mr ~ 50 000, equivalent to the S7 subunit of the proteasome (Dubiel et al, 1993) (Figure 2B), while there was no change in the expression of this protein in the hearts of PIF-treated animals (Figure 2C). There was a 2.8-fold increase in the functional activity of the proteasome, as determined by the chymotrypsin-like activity, in gastrocnemius muscle from PIF-treated mice, using the fluorogenic substrate succinylLLVY-MCA, while there was no significant change in heart muscle (Figure 2D). These results confirm that PIF upregulates the proteasome proteolytic pathway only in skeletal muscle.

To determine whether PIF acted directly to invoke these changes in gene expression further studies were conducted employing $C_2C_{12}$ murine myoblasts, since PIF has been previously shown to enhance protein degradation in these cells as a result of increased production of 15-HETE (Smith et al, 1999). The release of [$^3$H] phenylalanine from prelabelled cells was used as an index of proteolysis. PIF produced a dose-related elevation of protein breakdown with a maximal effect at 2–4 nM (Figure 3), which was attenuated by a monoclonal antibody to PIF (Todorov et al, 1996b)

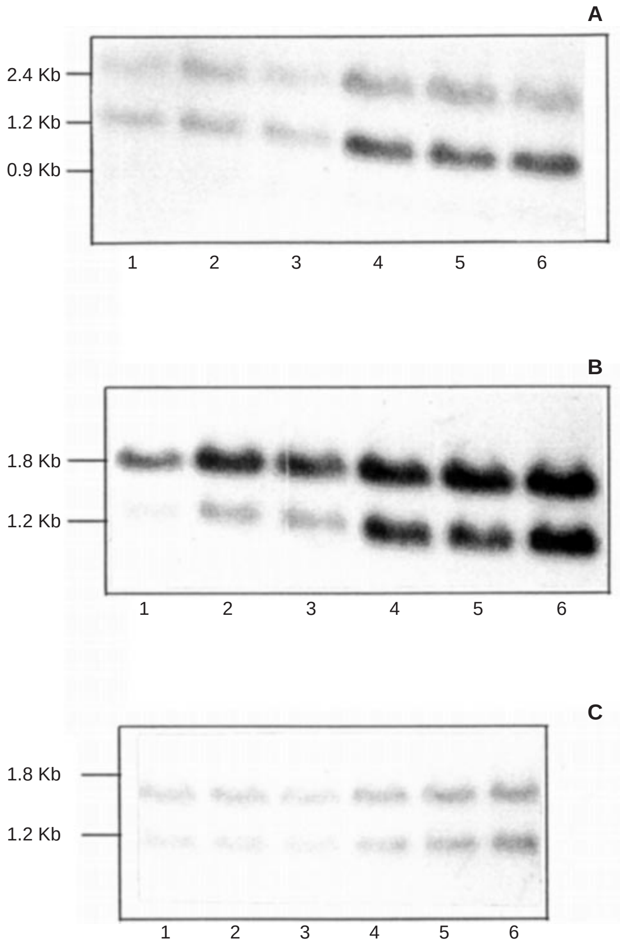

**Figure 1** Expression of mRNA for ubiquitin and 14KE2 in gastrocnemius muscle. (**A**) Ubiquitin, (**B**) 14KE2 ubiquitin carrier protein in gastrocnemius muscle and (**C**) heart from mice administered either PBS (lanes 1–3) or PIF (lanes 4–6) 24 h after the first injection. Kb (kilobase), size of the transcripts

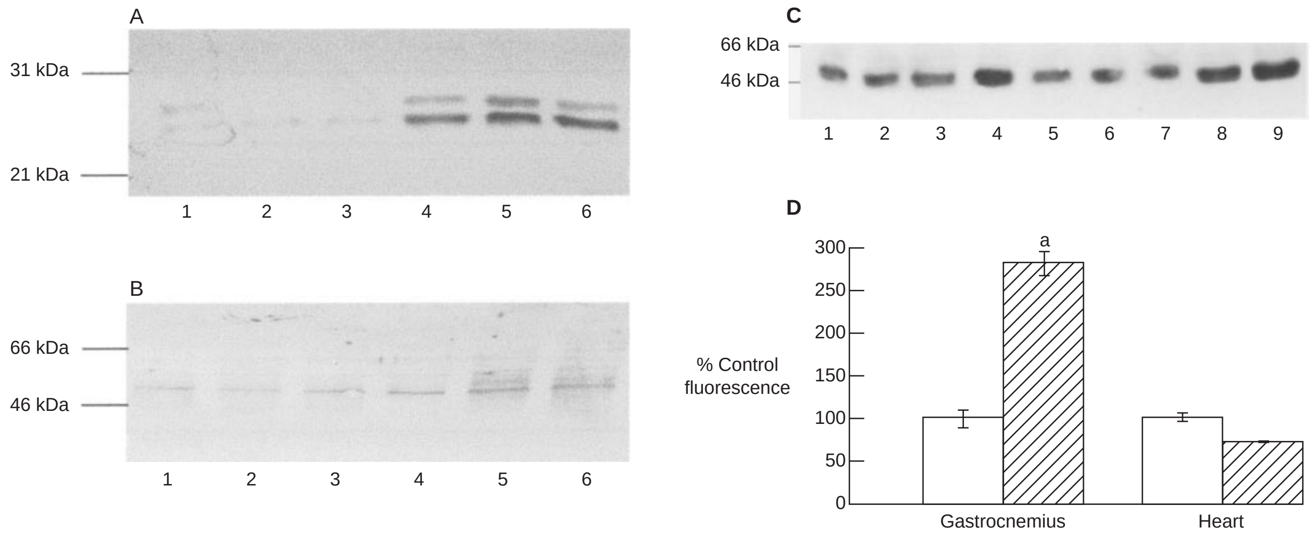

**Figure 2** Expression of proteasome subunits in gastrocnemius muscle. (**A**) Western blot analysis of soluble extracts of gastrocnemius muscles of mice 24 h after administration of PBS (lanes 1–3) or PIF (lanes 4–6) fractionated on 10% SDS-polyacrylamide gels and detected with rabbit polyclonal antibody to the 20S proteasome core (Affiniti Research Products, Exeter, UK) at a dilution of 1:1000. (**B**) As above but detected with mouse anti-human MSSI antibody at a dilution of 1:1000. (**C**) Western blot analysis of soluble extracts of heart muscle after administration of either PBS (lanes 1–4) or PIF (lanes 5–9) detected with mouse anti-human MSS1 antibody. Development was by an ECL system (Amersham, UK) using either peroxidase conjugated goat anti-rabbit Ig, (**A**) or sheep anti-mouse Ig, (**B**) and (**C**) (**D**) chymotryptic activity of soluble extracts of gastrocnemius muscle and heart from PIF-treated (hatched boxes) and control (open boxes) mice. The results are expressed as mean ± SEM where $n = 5$. Differences from control are expressed as a, $P = 0.008$ as determined by Student's *t*-test

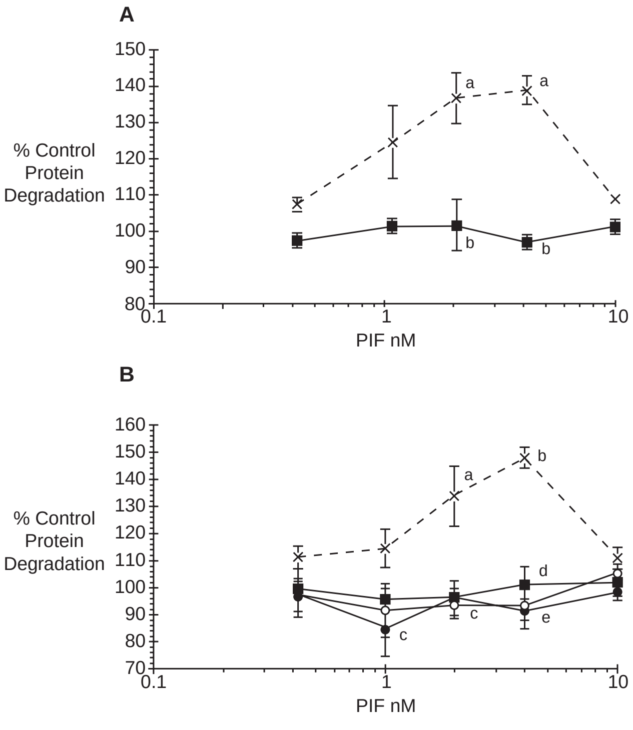

**Figure 3** Effect of murine anti-PIF monoclonal antibody and the proteasome inhibitor MG115 on PIF-induced protein catabolism. (**A**) $C_2C_{12}$ myoblasts were incubated with PIF alone (x) or with anti-PIF Ig (10 $\mu$gml$^{-1}$) (■) and protein degradation was determined after 24 h as in methods. (**B**) $C_2C_{12}$ myoblasts were incubated with PIF alone (x) or in the presence of MG115 at 1 (●), 10 (○) and 50 (●) $\mu$M and protein degradation was determined after 24 h. Results are shown as mean ± SEM, where $n = 9$. Differences from control cultures incubated in the absence of PIF are indicated as a, $P = 0.05$ and b, $P < 0.01$, while differences from PIF-treated cells are indicated as c, $P < 0.05$, d, $P < 0.01$ and e, $P < 0.005$ using 2-way ANOVA followed by Tuckey's test

(Figure 3A), and the reversible proteasome inhibitor benzyloxy-carbonyl-Leu-Leu-L-norvalinal (MG115) (Lee and Goldberg, 1996) at concentrations between 1 and 50 μM (Figure 3B). The effect was completely inhibited by the highly specific and irreversible proteasome inhibitor lactacystin, which does not inhibit lysosomal protein degradation in the cell (Fenteany and Schreiber, 1998), at a concentration of 10 μM. Glycerol gradient analysis of soluble extracts of $C_2C_{12}$ cells showed chymotrypsin-like enzyme activity in fractions 5–12, which was elevated in PIF-treated cells (Figure 4A). Western blot analysis of these fractions using anti-MSSI antibody, confirmed the presence of a protein of Mr ~ 50 000 (24) (Figure 4B). Western blot analysis of $C_2C_{12}$ cells treated with PIF showed an increased expression of MSSI at concentrations as low as 1.0 nM (Figure 4C), with further increases up to 4 nM, after which there was a decrease in expression with further concentrations of PIF. Thus the dose–response curve for induction of proteasome expression by PIF correlated with that for protein degradation (Figure 4), confirming that PIF acts to directly stimulate the proteasome pathway in $C_2C_{12}$ cells in vitro.

### DISCUSSION

There are 3 major proteolytic pathways involved in the intracellular degradation of proteins, the lysosomal pathway, $Ca^{2+}$-activated proteinases and the ubiquitin-proteasome system. Lysosomal proteolysis plays a minor role in skeletal muscle protein breakdown (Attaix and Taillander, 1998), while the $Ca^{2+}$-dependent proteinases may be qualitatively important for the degradation of specific, but quantitatively minor proteins. In contrast the ATP-ubiquitin-dependent pathway plays an important role in muscle protein degradation induced by starvation (Wing and Goldberg, 1993), sepsis (Voisin et al, 1996), metabolic acidosis (Mitch et al, 1994), denervation atrophy (Medina et al, 1995), head trauma (Mansoor et al, 1996) and cancer cachexia, both in animal models (Temparis et al, 1994; Lorite et al, 1998),

© 2001 Cancer Research Campaign

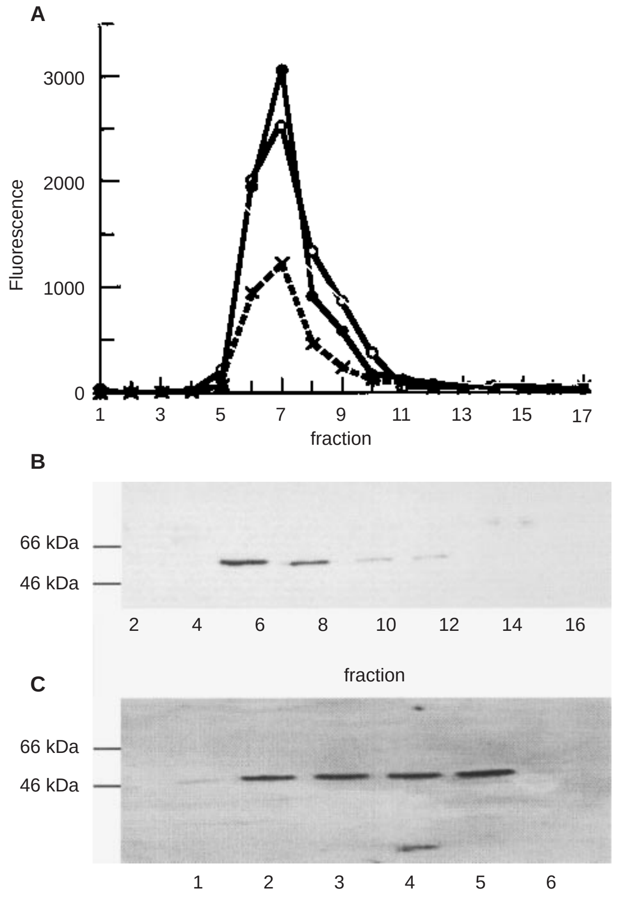

**Figure 4** (**A**) Separation and expression of proteasomes. Glycerol gradient analysis of proteasomes from $C_2C_{12}$ cells either untreated (x), or treated with PIF at 2 (○) or 4 (●) nM for 24 h. Proteolytic activity was separated from the main protein peak. The data have been normalized to take account of variations in protein content of the individual fractions. (**B**) Western blot analysis of fractions from the glycerol gradient in A (2.4 μg protein) fractionated on 10% SDS-polyacrylamide gels and detected with anti-human MSSI at a 1:1000 dilution. Detection was by an ECL system (Amersham, UK) using peroxidase conjugated sheep anti-mouse 1 g. (**C**) Western analysis of unfractionated supernatants of $C_2C_{12}$ myoblasts, performed as in (**B**) 24 h after addition of either PBS (lane 1) or 10 (lane 2), 4.0 (lane 3), 2.0 (lane 4), 1.0 (lane 5) or 0.5 (lane 6) nM PIF. The densitometric values are (arbitrary units), lane 1, 12; lane 2, 82; lane 3, 106; lane 4, 113; lane 5, 130 and lane 6, 41

and cancer patients (Attaix and Taillander, 1998). In this process ubiquitin is first activated by a ubiquitin-activating enzyme (E1), which then transfers the ubiquitin to a carrier protein (E2), which either ligates the ubiquitin directly to the target protein, or does so in the presence of a ubiquitin-protein ligase (E3). Conjugation mediated by E2 has been suggested to be a rate-limiting step in the pathway (Wing and Banville, 1994).

Previous in vivo studies with PIF have shown an energy requirement for proteolysis and an increase in ubiquitin-protein conjugates in gastrocnemius muscle (Lorite et al, 1998). The present study has provided evidence for an enhanced expression of components of the ATP-ubiquitin-dependent proteolytic pathway within 24 h of administration of PIF. Thus Northern blots showed mRNA levels for ubiquitin, the Mr 14 000 ubiquitin-conjugating enzyme (E2), as well as the proteasome subunit C9, to be all significantly increased in gastrocnemius muscle. Such changes occur rapidly, and in animals with normal food intake, despite the loss of body weight, and in a coordinate manner, as they do in experimental models of cancer cachexia (Temparis et al, 1994; Lorite et al, 1998). The Mr 14 000 E2 species best supports E3-dependent conjugate formation and protein breakdown (Wing and Banville, 1994). 2 mRNA transcripts of 1.2 and 1.8 kb were detectable arising from different sites of polyadenylation. Of the 2, the 1.2 kb transcript showed the major elevation after PIF administration. The 1.2 kb transcript has also been reported to be increased in starvation (Wing and Banville, 1994), sepsis (Voisin et al, 1996), dexamethasone treatment (Dardevet et al, 1995) and experimental cancer cachexia (Temparis et al, 1994; Lorite et al, 1998), while the 1.8 kb transcript is not significantly changed. The relevance of this elevation of the 1.2 kb transcript to protein degradation induced by PIF is shown by the lack of an enhanced expression in both heart and liver (not shown) for which wasting is not observed (Lorite et al, 1998). An enhanced proteasome activity was also observed, together with increased expression of the 20S proteasome core and the 19S regulatory subunit. Increased gene expression of proteasomal subunits has been suggested (Temparis et al, 1994) to be rate-limiting for protein breakdown.

These changes in expression of the components of the ATP-ubiquitin-dependent proteolytic pathway arise from a direct effect of PIF and are not due to the induction of intermediary facilitators in the host. Thus $C_2C_{12}$ myoblasts in vitro responded to PIF by an increase in protein degradation, which was dose-related and inhibited by higher concentrations of PIF (Smith et al, 1999). This was associated with an increase in the chymotryptic activity of the 26S proteasome, and an increase in an ATPase, MSSI, equivalent to the S7 subunit of the proteasome (Dubiel et al, 1993). In addition the proteasome inhibitors MG115 and lactacystin completely attenuated protein degradation induced by PIF, confirming the relevance of activation of the proteasome pathway to catabolism of muscle proteins.

Few other agents are known to activate this pathway directly. Enhanced skeletal muscle proteolysis in rats after tumour necrosis factor-α (TNF-α) administration is associated with an increased gene expression and higher levels of free and conjugated ubiquitin (Garcia-Martinez et al, 1993). This has been attributed to a direct effect of TNF-α since incubation of isolated rat soleus muscle with TNF-α for 180 min caused an increase in ubiquitin gene expression, although there was no change in the expression of the C8 proteasome subunit (Llovera et al, 1997). However, care must be taken in interpreting these results to mean an activation of the ATP-ubiquitin-dependent proteolytic pathway, because ubiquitin has roles other than in proteolysis (St John et al, 1986). Using $C_2C_{12}$ myotubes as a model of muscle Ebisui et al (1995) showed TNF-α to have no effect on the mRNA level of subunits C2 and C8 of the 20S proteasome and subunit S4 of the 26S proteasome, while interleukin-6 (IL-6) caused an increased expression of all subunits and reduced the half-life of long-lived proteins.

The signals mediating the response of PIF to the coordinate upregulation of ubiquitin, E2 and proteasome subunits are unknown, but could involve metabolites of arachidonic acid. We have shown (Smith et al, 1999) that PIF produced an increased release of arachidonic acid from $C_2C_{12}$ myoblasts that was blocked by eicosapentaenoic acid, an inhibitor of protein degradation. The arachidonic acid was metabolized to prostaglandins and hydroxyeicosatetraenoic acids (HETE), but only 15-HETE produced a significant increase in protein degradation. Further studies will attempt to elucidate the mechanisms involved.

*© 2001 Cancer Research Campaign*

### ACKNOWLEDGEMENTS

This work was supported by the Association for International Cancer Research (AICR). We thank Mr M Wynter for the tumour transplantations and for the intravenous injections of the animals.

### REFERENCES

Attaix P and Taillander D (1998) The critical role of the ubiquitin-proteasome pathway in muscle wasting in comparison to lysosomal and $Ca^{2+}$-dependent systems. *Adv Mol Cell Biol* **27**: 235–266

Beck SA and Tisdale MJ (1987) Production of lipolytic and proteolytic factors by a murine tumor-producing cachexia in the host. *Cancer Res* **47**: 5919–5923

Cariuk P, Lorite MJ, Todorov PT, Field WN, Wigmore SJ and Tisdale MJ (1997) Induction of cachexia in mice by a product isolated from the urine of cachectic cancer patients. *Br J Cancer* **76**: 606–613

Chomczynski P and Sacchi N (1987) Single step method of RNA isolation by acid guanidinium thiocyanate-phenol-chloroform extraction. *Anal Biochem* **162**: 156–159

Dardevet D, Sornet C, Taillandier D, Savary I, Attaix D and Grizard J (1995) Sensitivity and protein turnover response to glucocorticoids are different in skeletal muscle from adult and old rats. Lack of regulation of the ubiquitin-proteasome pathway in aging. *J Clin Invest* **96**: 2113–2119

Dawson SP, Arnold JE, Mayer NJ, Reynolds SE, Billet MA, Gordan C, Collenux L, Kloetzel PM, Tanaka K and Mayer RJ (1995) Developmental changes of the 26S proteasome in abdominal intersegmental muscles of Manduca sexta during programmed cell death. *J Biol Chem* **270**: 1850–1858

Dubiel W, Ferrell K and Rechsteinier M (1993) Peptide sequence identifies MSSI, a modulator of HIV Tat-mediated transactivation as subunit 7 of the 26S protease. *FEBS Lett* **323**: 276–278

Ebisui C, Tsujinaka T, Morimoto T, Kan K, Iijma S, Yano M, Kominami E, Tanaka K and Monden M (1995) Interleukin-6 induces proteolysis by activating intracellular proteases (cathepsins B and L, proteasome) in C2C12 myotubes. *Clin Sci* **89**: 431–439

Eden E, Ekman L, Bennegard K, Lindmark L and Lundholm K (1984) Whole body tyrosine flux in relation to energy expenditure in weight-losing cancer patients. *Metabolism* **33**: 1020–1027

Fearon KCH, Hansell DT, Preston T, Plumb JA, Davies J, Shapiro D, Shenkin A, Calman KC and Burns HJG (1988) Influence of whole body protein turnover rate on resting energy expenditure in patients with cancer. *Cancer Res* **48**: 2590–2595

Fearon KCH, McMillan DC, Preston T, Winstanley FP, Cruickshank AM and Shenkin A (1991) Elevated circulating interleukin-6 is associated with an acute phase response but reduced fixed hepatic protein synthesis in patients with cancer. *Ann Surg* **213**: 26–31

Fenteany G and Schreiber SL (1998) Lactacystin, protesome function, and cell fate. *J Biol Chem* **273**: 8545–8548

Garcia-Martinez C, Agell N, Llovera M, Lopez-Soriano FJ and Argiles JM (1993) Tumor necrosis factor-$\alpha$ increases the ubiquitinization of rat skeletal muscle proteins. *FEBS Lett* **323**: 211–214

Heber D, Chlebowski RT, Ishibashi PE, Herrold JN and Black JB (1982) Abnormalities in glucose and protein metabolism in non-cachectic lung cancer patients. *Cancer Res* **42**: 4815–4819

Kumatori A, Tanaka K, Tamura T, Fujiwara T, Ichihara A, Tokunaga F, Onikura A and Iwanaga S (1990) cDNA cloning and sequencing of component C9 of proteasomes from rat hepatoma cells. *FEBS Lett* **264**: 279–282

Lee DH and Goldberg AL (1996) Selective inhibitors of the proteasome-dependent and vacuolar pathways of protein degradation in Saccharomyces cerevisiae. *J Biol Chem* **271**: 27280–27285

Llovera M, Garcia-Martinez C, Agell N, Lopez-Soriano FJ and Argiles JM (1997) TNF can directly induce the expression of ubiquitin-dependent proteolytic system in rat soleus muscles. *Biochem Biophys Res Commun* **230**: 238–241

Lorite MJ, Thompson MG, Drake JL, Carling G and Tisdale MJ (1998) Mechanism of muscle protein degradation induced by a cancer cachectic factor. *Br J Cancer* **76**: 850–856

Lundholm K, Bylund AC, Holm J and Schersten T (1976) Skeletal muscle metabolism in patients with malignant tumour. *Eur J Cancer* **12**: 465–473

Mansoor O, Beaufrere B, Boirie Y, Ralliere C, Taillandier D, Aurousseau E, Schoeffler P, Arnal M and Attaix D (1996) Increased mRNA for components of the lysosomal, $Ca^{2+}$-activated and ATP-ubiquitin-dependent proteolytic pathways in skeletal muscle from head trauma patients. *Proc Natl Acad Sci USA* **93**: 2714–2718

Medina R, Wing SS and Goldberg AL (1995) Increase in levels of polyubiquitin and proteasome mRNA in skeletal muscle during starvation and denervation atrophy. *Biochem J* **307**: 631–637

Mitch WE, Medina R, Grieber S, May RC, England BK, Price SR, Bailey JL and Goldberg AL (1994) Metabolic acidosis stimulates muscle protein degradation by activating the adenosine triphosphate-dependent pathway involving ubiquitin and proteasomes. *J Clin Invest* **93**: 2127–2133

O'Keefe SJD, Ogden J, Ramjee G and Rund J (1990) Contribution of elevated protein turnover and anorexia to cachexia in patients with hepatocellular carcinoma. *Cancer Res* **50**: 1226–1230

Orino E, Tanaka K, Tamura T, Sone S, Ogura T and Ichihara A (1991) ATP-dependent reversible association of proteasomes with multiple protein components to form 26S complexes that degrade ubiquitinated proteins in human HL-60 cells. *FEBS Lett* **284**: 206–210

Preston T, Fearon KCH, Robertson I, East BW and Calman KC (1987) Tissue loss during severe wasting in lung cancer patients. In Vivo Body Composition Studies, (Ellis KJ, Yasumara S and Morgan WD) (eds) pp. 60–69. *Institute of Physical Sciences*: London

Smith HJ, Lorite MJ and Tisdale MJ (1999) Effect of a cancer cachectic factor on protein synthesis/degradation in murine $C_2C_{12}$ myoblasts – Modulation by eicosapentaenoic acid. *Cancer Res* **59**: 5507–5513

St John T, Gallatin WM, Siegelman M, Smith HT, Friel VA and Weisman IL (1986) Expression cloning of a lymphocyte homing receptor cDNA: ubiquitin is the reactive species. *Science* **231**: 845–850

Temparis S, Asensi M, Taillandier D, Aurousseau E, Larbaud D, Obled A, Béchet D, Ferrara M, Estrela JM and Attaix D (1994) Increased ATP-ubiquitin-dependent proteolysis in skeletal muscles of tumor-bearing rats. *Cancer Res* **54**: 5568–5573

Todorov P, Cariuk P, McDevitt T, Coles B, Fearon K and Tisdale M (1996a) Characterization of a cancer cachectic factor. *Nature* **379**: 739–742

Todorov PT, McDevitt TM, Cariuk P, Coles B, Deacon M and Tisdale MJ (1996b) Induction of muscle protein degradation and weight loss by a tumor product. *Cancer Res* **56**: 1256–1261

Voisin L, Breville D, Combaret L, Pouyet C, Taillandier D, Aurousseau E, Obled C and Attaix D (1996) Muscle wasting in a rat model of long-lasting sepsis results from the activation of lysosomal, $Ca^{2+}$-activated and ubiquitin-proteasome proteolytic pathways. *J Clin Invest* **97**: 1610–1617

Windsor JA and Hill GL (1988) Risk factors for postoperative pneumonia. The importance of protein depletion. *Ann Surg* **298**: 209–217

Wing SS and Goldberg AL (1993) Glucocorticoids activate the ATP-ubiquitin-dependent proteolysis system in skeletal muscle during fasting. *Am J Physiol* **264**: E669–E676

Wing SS and Banville D (1994) 14-kDa ubiquitin-conjugating enzyme: structure of the rat gene and regulation upon fasting and by insulin. *Am J Physiol* **267**: E39–E48

 *© 2001 Cancer Research Campaign*
